# An evaluation of prescribing practices for community-acquired pneumonia (CAP) in Mongolia

**DOI:** 10.1186/1472-6963-13-379

**Published:** 2013-10-03

**Authors:** Gereltuya Dorj, Delia Hendrie, Richard Parsons, Bruce Sunderland

**Affiliations:** 1 School of Pharmacy, Curtin University, Bentley, Western Australia, Australia; 2 Centre of Population Health Research, Curtin Health Innovation Research Institute, Bentley, Western Australia, Australia

## Abstract

**Background:**

Community-acquired pneumonia (CAP) is a significant cause of morbidity and mortality in all age groups worldwide. It may be classified as mild/moderate or severe, the latter usually requiring hospitalisation. Although, there are many studies reported in relation to CAP, there is relatively little known about the treatment of CAP and its antibiotic use in Mongolia. The study aim was to evaluate prescribing practices for the treatment of mild/moderate CAP in Mongolia with respect to national prescribing guidelines.

**Methods:**

Written prescriptions with a written diagnosis of CAP included were collected prospectively and sequentially for ten weeks from a purposefully selected sample of community pharmacies in rural and urban areas of Mongolia. The data collected included the patient’s age, gender, medication details, frequency and number of doses prescribed. Evaluation was with respect to the Mongolian Standard Treatment Guidelines (2005, 2008). Statistical differences between groups were tested using the Chi-squared and Fisher’s exact tests.

**Results:**

Prescriptions were collected from 22 pharmacies and represented the prescribing practices of 118 doctors. The study enrolled 394 (193 adults and 201 children) patients, with a median age for children of 2.0 years (range: 0.03-12) and adults of 33.0 years (range: 13–92).

The most commonly prescribed drugs were aminopenicillins, vitamins, and mucolytics, with the median number of drugs being three per prescription. Inappropriate drug selection was similar for adults (57.7%) and children (56.6%), and the major reason for an overall frequency of inappropriate prescribing for adults was 89.0% and for children 78.0%. Doctors in urban areas prescribed more inappropriate drugs than those in rural areas for both children and adults, *p* = .0014. The proportion of prescribed injections was 28.4% for adults and 9.0% for children, and for adults was significantly higher in urban areas. The prescribing standard for non-hospitalized patients in Mongolia states that injections should not be prescribed.

**Conclusions:**

The high level of inappropriate prescribing for mild/moderate CAP highlights the need to develop comprehensive and reliable procedures nationwide to improve prescribing practices in Mongolia.

## Background

Community-acquired lower respiratory tract infection (LRTI) is a common cause of acute illness both in developing and developed countries. The spectrum of the disease ranges from a mild mucosal colonisation or infection, acute bronchitis or acute exacerbation of chronic bronchitis/chronic obstructive pulmonary disease, to overwhelming symptoms in the patient presenting with severe community-acquired pneumonia. Pneumonia is broadly classified into two categories: community-acquired (CAP) and hospital-acquired. CAP is a significant cause of morbidity and mortality in all age groups, especially the elderly [1], which is a patient population group that continues to grow. Death rates associated with CAP have not changed greatly, partly because of increased numbers of patients with comorbidity and patients at risk [2].

Rational drug use occurs when an appropriate drug is prescribed and administered according to the appropriate dosage regimen and the drug should be affordable, available, dispensed correctly, in correct doses at adequate time intervals [3]. The basis for achievement of rational drug use is conformity with standard treatment guidelines. Moreover, rational dispensing correlates with drug supply procedures and also the competency and knowledge of the health care provider. The World Health Organisation (WHO) was the first to launch a major step (Model Essential Drug List) towards the rational use of drugs in 1977 [3]. This list was designed to help countries to develop their own national lists. Later, the International Network for the Rational Use of Drugs (INRUD) was established to conduct multidisciplinary research to promote the rational use of drugs [4].

Common examples of irrational drug use include the prescribing of antibiotics for viral infections and excessive or unnecessary use of injections. The consequences of irrational drug use include poor or limited quality of care [5,6], high cost of therapy [7,8] and increased incidence of adverse effects such as prolonged morbidity, mortality, drug toxicity, hospitalization for longer periods, antibiotics resistant to microorganisms and the associated infections [9,10]. Antibiotics are one of the most commonly used treatments in modern medicine [11]. Excessive usage of antibiotics can be costly, and may have a large cost impact when there is a limited drug budget. Although it is difficult to estimate, the global sale of antibiotics is reported as 6 to 21% of the pharmaceutical market, 3 to 25% of total prescriptions [12], and 15 to 30% of drug expenditure [13].

As recommended by the Australian Commission on Safety and Quality in Healthcare, antimicrobial use should be optimised by managing through a number of interventions, often referred to as antimicrobial stewardship programs [14]. An essential core to implement antimicrobial stewardship programs is monitoring of prescribing with respect to the guidelines on appropriate use of antibiotics, including information regarding appropriate selection, dosing, route, and duration of antimicrobial therapy [15]. Other interventions include the restriction of selected antibiotics and “stop-orders” after predetermined time periods. The goals of an antimicrobial stewardship program include optimization of clinical outcomes while minimizing unintended consequences of antimicrobial use such as toxicity, and the emergence of resistance. Moreover, it is aimed to reduce unnecessary costs associated with health care [16].

At present, clinical guidelines are widely available in many countries [17,18]. These guidelines should consider different risk factors, such as age, comorbidity and initial clinical severity [19] and there should be evidence-based implementation strategies at a local level in each country.

In accordance with WHO initiatives, the National Essential Drug List of Mongolia was first published in 1991 and it has subsequently been revised five times [20]. Generic drugs are promoted by the Government through its existing legislation. CAP is a significant disease that requires urgent appropriate management, including antibiotics. There is relatively little known about the treatment of mild/moderate CAP in Mongolia, and in particular, the drugs used. This study is the first community-based assessment of the treatment of non-hospitalized patients with mild/moderate CAP in Mongolia. The primary aim of this study was to evaluate the appropriateness of prescribing practices for mild/moderate CAP based on the criteria established by the standard treatment guidelines in Mongolia. A secondary objective was to investigate the extent of injections prescribed for mild/moderate CAP in Mongolia.

## Methods

### Data collection

Prescriptions submitted to community pharmacies in Mongolia with only a diagnosis of mild/moderate CAP written on the prescription by the prescriber, were collected prospectively and sequentially. According to the National Guideline for Good Prescribing and Dispensing Practice of Mongolia (Regulations), all physicians must record the diagnosis on the prescription. A data collection form was developed and translated from English to Mongolian and back-translated into English, in order to assure the accuracy of data collection. All prescribed drugs, including their dosage, duration, route of administration and demographic information of patients were extracted from the prescriptions. Each drug was evaluated for rational prescribing based on the Standard Treatment Guidelines of Mongolia (2005, 2008) [21,22]. Appropriateness was assessed for each of the following indicators: drug selection, dosage form, single dose, frequency, prescribed quantity and prescribed duration. A drug was classified as “inappropriate” if any one of these indicators were not in accordance with the standard treatment guidelines. The assessment was based on a sequential cascading down effect, e.g. if first indicator was “inappropriate” then drug would be excluded from further analysis and would not appear in the second indicator, etc.

### Site selection

Mongolia, a developing country located between Russia and China, is one of the most sparsely populated countries in the world, with a total population of 2.75 million. It is divided into 21 provinces (aimag) and the capital city is Ulaanbaatar. About 40% of the population lives in the capital city.

According to the “National Standard Requirement for Pharmacy” [23], a main community pharmacy can have up to two branches. The main pharmacy can only be owned by pharmacists, but the pharmacy branch can be owned by a pharmacist or pharmacy technician. According to the latest statistics, there were 543 community pharmacies [24]. In addition, 302 remote pharmacies (soum) were registered as Revolving Drug Funds (RDFs) in 2011, provided by the government [24]. These can be managed by pharmacists or pharmacy technicians.

The site selection was based on the WHO Operational package for assessing, monitoring and evaluating country pharmaceutical situations [25]. The principle of selecting private pharmacies in the city and in each province was to select the closest private pharmacy to each public health facility surveyed. However, branches and RDFs were excluded in this study because branches of the pharmacies are legally restricted to Over the Counter (OTC) drugs and due to limited budget.

A convenience selection method was applied for pharmacies in rural areas based on discussion with local professionals. The sites selected were privately owned community pharmacies in towns in eight provinces (Bayankhongor, Bulgan, Govi-Altai, Khovsgol, Ovorkhangai, Sukhbaatar, Tuv, Uvs) and the remainder in the capital city (Ulaanbaatar).

### Period of the study

The study was conducted in the winter season (mean temperature -25°C) over a period of 10 weeks, from January until March, 2010, which is a period with a high prevalence of acute respiratory tract infections.

The study protocol was approved by the Human Research Ethics Committee, Curtin University, Western Australia (PH-11-2010).

### Data analysis

All data were entered and analysed using SPSS software (version 18.0). The drugs prescribed for the diagnosis of mild/moderate CAP were analysed against recommendations included in the Standard Treatment Guidelines for CAP (2005, 2008) and the National Guidelines for Good Prescribing Practice of Mongolia (Table 1). Decisions regarding appropriateness were made separately by two of the authors (GD and validated by BS). Differences were resolved by consensus. Differences in prescribing practices between adults and children and urban and rural areas were tested for statistical significance using the Chi-square statistic and Fisher’s Exact test. A p-value < 0.05 was taken to indicate a statistically significant association.

**Table 1:** Standard treatment guidelines for the management of mild/moderate CAP in Mongolia

**Adults**	**Mild/ moderate CAP**	
**Mongolian Standard Treatment Guidelines for Common Diseases: Pneumonia (2005)**	Oral amoxicillin (ampicillin) 500 mg every 6 hours, or erythromycin 500 mg every 6 hours	
**Children**		
**Treatment Guidelines for Common Diseases in Children: Pneumonia (Mongolian Standard) MNS 5836:2008**	Infants: Benzylpenicillin, aminoglycoside (gentamicin) injectionUp to five years old: Semi-synthetic penicillin (50 mg/kg/4 times) + gentamicin 7.5 mg/kg/once)-injection If available chloramphenicol (75 mg/kg/3 times a day) *Additional option: Cephalosporin II-III	If considered necessary, any of the following could be prescribed: Salbutamol, euphyllin, epinephrine Prednisolone, dexamethasone Vitamin C, A or E

Data analysis of the prescribing practices for children was limited by the lack of information in the guidelines for children aged 6–15 year old (Table 1).

## Results

### Study sites

Thirty pharmacies consisting of 20 in the Ulaanbaatar area and 10 in eight of the provinces were selected for inclusion in the study, of which 22 consented. This represented a response rate of 73%. All pharmacies that did not consent were in the city area stating they were too busy to participate.

### Selection and characteristics of participants

The study enrolled 394 (193 adults and 201 children) participants who were diagnosed with mild/moderate CAP. The prescriptions represented the prescribing practices of 118 doctors.

Table 2 shows the demographic characteristics of participants. The proportions of adults (48.9%) and children (51.0%) were almost equally represented,, with a median age for children of 2.0 years (range: 0.03-12) and adults of 33.0 years (range: 13–92). Male adults (49.7%) were in similar numbers to females (50.3%).

**Table 2:** Demographic characteristics of participants

**Characteristics**	**Number (%)**	**Gender**	**Median age (years)**	**Median weight (kg)**	**Location**
Adults	193 (48.9)	F=97 (50.3%)	33.0	-	City=124 (64.2%)
					Rural=69 (35.8%)
Children	201	F= 98 (48.8%)	2.0	13.7	City=111 (55.2%)
	(51.0)				Rural=90 (44.8%)
Total					394 (100%)

### Prescribing pattern of doctors

A total of 1100 drugs were prescribed for the 394 participants, with the most commonly prescribed being aminopenicillins (10.4% for adults and 18.3% for children), followed by vitamins, mucolytics (bromhexine), ciprofloxacin and paracetamol (Table 3).

**Table 3:** Most commonly prescribed drugs for patients with mild/moderate CAP

**Drug name**	**Prescribed frequency (N=1100)**	**Percentage (%)**	**ATC Code**
Aminopenicillins	163	16.0	J01CA04
Vitamin C	67	8.8	A11GA01
Bromhexine (Mucolyitic)	62	5.6	R05CB02
Paracetamol	57	3.5	N02BE01
Ciprofloxacin	52	4.7	J01MA02
Salbutamol	37	3.4	R03CC02
Erythromycin	36	3.3	J01FA01
Cotrimoxazole	34	2.7	J01EE01
Ketotifen (Antihistamine)	33	3.0	R06AX17
Calcium gluconate	32	2.9	A12AA03
Cefazoline	31	2.8	J01DB04
Sodium chloride	31	2.8	A12CA01
Chlorphenamine	29	2.6	R06AB04
Chitamon^a^	23	2.1	Herbal
Vitamin B Complex	17	1.6	A11EA

^a^Local product containing Glycyrrhiza uralensis Fisch, Thermopsis dahurica Czefr.

There was a low level of poly-pharmacy with the median number of drugs being three per prescription. There was no significant difference in the number of drugs prescribed for adults (p=0.63) and children, or in urban and rural locations (*p* = .98) (Table 4).

**Table 4:** Number of drugs prescribed per prescription

**Category**	**Adults**		**Children**	
	**Urban**	**Rural**	**Urban**	**Rural**
No. of patients	124	69	111	90
No. of prescribed drugs	368	188	301	243
Min	1.00	1.00	1.00	1.00
Median	3.00	3.00	3.00	3.00
Max	7.00	6.00	7.00	6.00
Mean	2.99	2.72	2.71	2.73
Std Dev	1.20	0.87	1.12	0.91
p value*	=0.63		=0.98	

*p-value was calculated based on number of patients vs. number of drugs in different areas.

### Frequency of inappropriate prescribing

**Figure 1:**
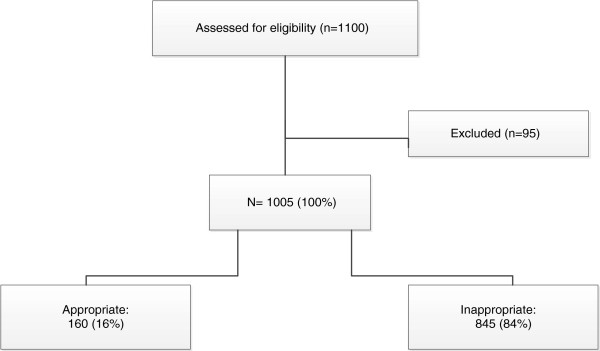
Appropriateness level of prescribing for patients with mild/ moderate CAP.

**Figure 2:**
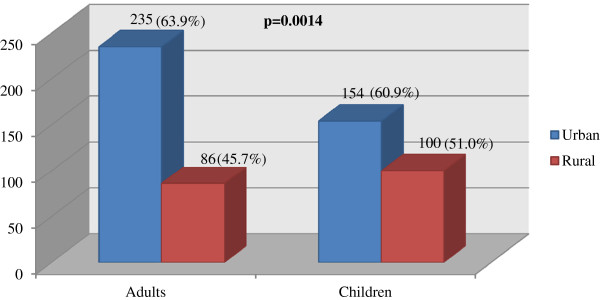
Inappropriateness levels of drug selection for patients with mild/ moderate CAP in urban and rural areas (y axis refers to number of drugs prescribed).

The overall level of inappropriate prescribing for all patients based upon application of the standard treatment guidelines was 845 (84.0%) (Figure 1). A total of 95 were not assessable because of lack of information about drug selection, dosage form, dose, frequency and duration in the current guidelines for children aged between 6 to 15 years (Figure 2).

The results of the assessment of prescription categories for patients with mild/moderate CAP are shown for children and adults in Tables 5 and 6 respectively. A chi-squared analysis showed a statistically significant difference between inappropriate prescribing for adults and children, χ [2] (1, n=1100) =22.8, *p* < .001. Relatively more adults were prescribed inappropriate drugs, largely as a result of the dosage frequency prescribed.

**Table 5:** Assessment of the prescriptions for children with mild/moderate CAP*

**Category**	**Drug selection**	**Dosage form**	**Dose**	**Frequency**	**Final result**
	**n (%)**	**n (%)**	**n (%)**	**n (%)**	**n (%)**
A	195 (43.4)	171 (87.7)	102 (59.6)	99 (97.1)	99 (22.1)
IA	254 (56.6)	24 (12.3)^a^	(see below)	3 (2.9)	350 (78.0)
OPD	-	-	1 (0.6)	-	-
UPD	-	-	68 (39.8)	-	-
NAI	95	95	95	95	95
Total assessable	449	195	171	102	449
Total	544	290	266	197	544

A- Appropriate, IA- Inappropriate, NAI- No assessable guideline information, OPD- Overprescribed dose, UPD- Underprescribed dose.

^a^Includes the number of appropriately selected drugs from the previous column.

^*^If the first indicator was “inappropriate”, then the prescription item classification was “inappropriate” and this drug was excluded from further analysis and would not appear in the second indicator.

**Table 6:** Assessment of the prescriptions for adults with mild/moderate CAP*

**Category**	**Drug selection**	**Dosage form**	**Dose**	**Frequency**	**Final result**
	**n (%)**	**n (%)**	**n (%)**	**n (%)**	**n (%)**
A	235 (42.3)	192 (81.7)	120 (62.5)	61 (50.8)	61 (11.0)
IA	321 (57.7)	43 (18.3)^a^	-	59 (49.2)	495 (89.0)
OPD	-	-	18 (9.4)	-	-
UPD	-	-	54 (28.1)	-	-
Total	556	235	192	120	556

A- Appropriate, IA- Inappropriate, OPD- Overprescribed dose, UPD- Underprescribed dose.

^a^Includes the number of appropriately selected drugs in inappropriate dosage forms only.

^*^If the first indicator was “inappropriate”, then the prescription item classification was “inappropriate” and this drug was excluded from further analysis and would not appear in the second indicator.

Inappropriate drug selection was the major reason for inappropriate prescribing for patients with CAP, with the extent of inappropriate drug selection similar for children (56.6%) and adults (57.7%). Doctors in urban areas prescribed a higher frequency of inappropriate drugs than those in rural areas for the population studied, χ [2] (1, n=575) =10.25, *p* = .0014 (Figure 2).

### Prescribing level of injectables

The proportion of drugs (n=1100) prescribed as injections was 28.4% for adults and 9.0% for children. Prescribing of injectables was significantly higher for adults in urban areas compared with rural areas χ [2] (1, n=556)=21.7, DF=1, *p* = < .001, but the difference between urban and rural prescribing of injectables was not significant for children (Table 7). In the case of antibiotics, the proportion of injectables prescribed was 34.7% in the urban (83/239) and 18.5% in rural areas (31/168). Since the current guidelines for ambulatory care does not allow any use of injectables for outpatients with moderate/mild CAP, this use of injections is non-compliant with one of the current prescribing standards for Mongolia [26]. Moreover, gentamicin is recommended for the treatment of mild/moderate CAP for children and it was prescribed for outpatients with mild/moderate CAP. However, this is available only as injectable, so the guideline recommendation is non-compliant with the prescribing standard.

**Table 7:** Proportion of prescribed injections for participants with mild/moderate CAP

**Category**	**No. of injectables n (%)**^**a**^	**No. of non-injectables n (%)**^**a**^	**Total**	***p *** **Value**
Urban adults	128 (23.0)	240 (43.2)	368	< .001
Rural adults	30 (5.4)	158 (28.4)	188	
Urban children	32 (5.9)	269 (49.4)	301	.141
Rural children	17 (3.1)	226 (41.5)	243	

^a^Percentage of drugs.

## Discussion

This is the first study to explore prescribing practices for mild/moderate CAP in Mongolia by comparing drugs prescribed with respect to government initiated treatment guidelines. The study revealed only low levels of poly-pharmacy with the average number of drugs prescribed being three per patient. This is consistent with a previous assessment of prescribing practices of the pharmaceutical sector undertaken in 2009 [27].

High levels of inappropriate prescribing were identified when evaluation occurred with respect to government produced treatment guidelines for CAP in Mongolia, with 84% of drugs inappropriately prescribed. The major reason causing inappropriate prescribing for both adults and children was inappropriate drug selection.

In a South African study examining the adherence to treatment guidelines for CAP, empirical antibiotic treatment for severe CAP accorded with local guidelines for 14 patients (8%) only. The remaining 168 patients (92%) were given treatment that was inconsistent with the guidelines [28].

The evaluation of prescriptions indicated diverse prescribing practices for patients with CAP. In most cases (93.4%) at least one antibiotic was prescribed. Ciprofloxacin, cotrimoxazole and ketotifen (antihistamine) were most commonly prescribed. These were inconsistent with the current guidelines and showed a lack of practical adoption and implementation of the treatment guidelines. Possible explanations for these findings could include lack of knowledge, attitude and awareness of the guidelines. In addition, some prescribers under-prioritise the guidelines because they may feel time and financial pressures from external factors [29,30].

Different prescribing practices between rural and urban areas were observed, with doctors in rural areas prescribing more appropriately than their counterparts in urban areas. Anecdotal statements from local health professionals suggested this could relate to the smaller population and possibly a better control of prescribing and dispensing practice. Another possible reason may relate to rural practitioners being less accessible to visits from pharmaceutical companies or receiving their literature. There were reports from doctors and pharmacists about financial incentives from the pharmaceutical companies in the form of extra income from prescribing and dispensing their drugs. This practice is illegal in Mongolia; however it is still common in some countries [31-33].

Unfortunately about 8% of all drugs prescribed for children with CAP in this study could not be assessed due to non-existent information in the current guidelines for children aged 5 to 16 years. This indicates that evidence-based treatment of children with CAP cannot be assured in the absence of guidelines.

Treatment guidelines are an important component of assuring appropriate drug use [34] and for the decision making process in health care practice. The need for and importance of well-documented, standardized guidelines is recognized as essential for the successful treatment of CAP [35]. However, despite being promulgated by well-regarded institutions, often compliance with national guidelines for treatment of CAP is poor [36,37]. The implementation of guidelines depends on the clarity of the statement and adaptability into practice. In addition, guidelines need to be readily assessable and endorsed by senior practitioners. A number of interventions to improve the prescribing behaviour can be found from the literature. However, a Cochrane review indicated that there was insufficient evidence to support the choice of intervention [38]. While single interventions may be as effective as multiple ones due to existing health infrastructure in developed countries, multiple intervention packages have been shown to be more beneficial in less developed countries [34]. These intervention packages often include building infrastructure, such as supervisory systems, that are likely to increase their impact [34]. In addition, tailoring interventions to target specific barriers to compliance was reported to be effective in improving professional practice [39-41].

The use of unnecessary injections is a common occurrence in developing countries and it is widely recognized that unsafe healthcare injections can transmit HBV [42], HCV [43], HIV [44], viral haemorrhagic fever and other bloodborne pathogens [45]. In this study, the prescribing level of injections for the treatment of mild/moderate CAP was approximately 18% of all drugs. Moreover, inconsistency between guidelines was observed. Gentamicin is recommended in the current treatment guideline for children with CAP [21]. However, it is available only as an injection and this recommendation does not comply with the Good Prescribing and Dispensing Practice regulations of Mongolia [46]. According to a study of injection practices in Mongolia, the average number of injections was 13 per patient for one year in Mongolia in 2002, which was the highest rate of injection usage in the Western Pacific Region [47]. Most of the injections (antibiotics) were administered for the treatment of pneumonia. Regardless of the government’s effort to develop guidelines to promote and implement rational drug use, there are still problems with unnecessary and inappropriate use of injections in community settings and with the current guidelines.

### Limitations

The study has two main limitations. Firstly, the estimates were based on a one point in time observation completed in the winter period. Secondly, although the study aimed to provide a representative sampling in location and size, only about 4% of all main community pharmacies could be included. However it is the habits of prescribers that were assessed in this study. The study assessed the prescribing practice of 118 prescribers which represents 15% of all family group practitioners in Mongolia. Therefore, the results should be representative of the prescribing practice for the treatment of CAP at the national and rural levels.

## Conclusions

The study has shown unacceptably high levels of inappropriate prescribing for mild/moderate CAP in Mongolia and in addition has highlighted the inadequacy and inconsistency of the current treatment guidelines. The findings have important implications for the health status of patients being treated for mild/moderate CAP and suggest the need for appropriate interventions to be developed and introduced by authorities to address the issues raised.

## Competing interests

The authors declare that they have no competing interests.

## Authors’ contributions

GD carried out the study and drafted the manuscript. DH participated in the design and revised the manuscript. RP performed the statistical analysis and revised the manuscript. BS conceived of the study, and participated in its design and coordination and revised the manuscript. All authors read and approved the final manuscript.

## Pre-publication history

The pre-publication history for this paper can be accessed here:

http://www.biomedcentral.com/1472-6963/13/379/prepub
